# A fully coupled fluid-structure interaction model for patient-specific analysis of bioprosthetic aortic valve haemodynamics

**DOI:** 10.3389/fbioe.2025.1584509

**Published:** 2025-05-29

**Authors:** Zhongjie Yin, Chlöe Armour, Selene Pirola, Harkamaljot Kandail, Xiaoxin Kan, Pankaj Garg, Rui Li, Toufan Bahrami, Saeed Mirsadraee, Xiao Yun Xu

**Affiliations:** ^1^ Department of Chemical Engineering, Imperial College London, South Kensington Campus, London, United Kingdom; ^2^ National Heart and Lung Institute, Imperial College London, London, United Kingdom; ^3^ Department of BioMechanical Engineering, TU Delft, Delft, Netherlands; ^4^ Medtronic Neurovascular, Irvine, CA, United States; ^5^ Centre for Vascular Surgery and Wound Care, Jinshan Hospital, Fudan University, Shanghai, China; ^6^ Norwich Medical School, University of East Anglia, Norfolk, United Kingdom; ^7^ Norfolk and Norwich University Hospitals NHS Foundation Trust, Norfolk, United Kingdom; ^8^ Department of Cardiothoracic Surgery, Royal Brompton and Harefield Hospitals NHS Trust, London, United Kingdom; ^9^ Department of Radiology, Royal Brompton and Harefield Hospitals NHS Trust, London, United Kingdom

**Keywords:** bioprosthetic aortic valve, fluid-structure interaction, 4D flow, haemodynamics, wall shear stress

## Abstract

**Background:**

Bioprosthetic aortic valves (BPAV) have been increasingly used for surgical aortic valve replacement (SAVR), but long-term complications associated with structural valve deterioration remain a concern. The structural behaviour of the valve and its surrounding haemodynamics play a key role in the long-term outcome of SAVR, and these can be quantitively analysed by means of fluid-structure interaction (FSI) simulation. The aim of this study was to develop a fully coupled FSI model for patient-specific analysis of BPAV haemodynamics.

**Methods:**

Using the Edwards Magna Ease valve as an example, the workflow included reconstruction of the aortic root from CT images and the creation of valve geometric model based on available measurements made on the device. Two-way fully coupled FSI simulations were performed under patient-specific flow conditions derived from 4D flow magnetic resonance imaging (MRI), the latter also provided data for model validation.

**Results:**

The simulation results were in good agreement with haemodynamic features extracted from 4D flow MRI and relevant data in the literature. Furthermore, the FSI model provided additional information that cannot be measured *in vivo*, including wall shear stress and its derivatives on the valve leaflets and in the aortic root.

**Conclusion:**

The FSI workflow presented in this study offers a promising tool for patient-specific assessment of aortic valve haemodynamics, and the results may help elucidate the role of haemodynamics in structural valve deterioration.

## 1 Introduction

Aortic valve stenosis (AS) is one of the most common aortic valve diseases. It occurs in 12.4% of people aged over 75 (Santangelo et al., 2023), with a mortality rate of over 25% per year when a severe symptomatic AS is left untreated (Carabello and Paulus, 2009). Bioprosthetic aortic valves (BPAV) have been increasingly used for surgical replacement of the diseased aortic valve. One of the most widely used BPAV products is the Carpentier-Edwards Perimount valve series. It was first introduced in the United States in 1981, and its representative product is Edwards Magna Ease valve (Rajab et al., 2020). This product consists of three bovine pericardial leaflets fixed by a flexible cobalt-chromium alloy profile and attached to a silicone rubber sewing ring that is sutured onto the aortic valve annulus.

Clinical studies have demonstrated that the Magna Ease valve has good long-term durability as well as excellent haemodynamic performance (Banbury et al., 2002; Bourguignon et al., 2015; Rajab et al., 2020; Mayr et al., 2021; Minardi et al., 2011; Piperata et al., 2022). It was found that the mean transvalvular pressure gradient and effective orifice area (EOA) increased steadily during the first 10 years and after which they stabilised (Banbury et al., 2002). Additionally, the rate of freedom from reoperation due to structural valve deterioration (SVD) was 70.8% ± 4.1% after 15 years of implantation (Bourguignon et al., 2015). Nevertheless, SVD still affects a significant number of patients, which is mainly seen as thickening, calcification, or tearing of the prosthetic valve materials, which can ultimately lead to haemodynamic dysfunctions such as valve stenosis and/or regurgitation (Dvir et al., 2018). It has been suggested that haemodynamics surrounding the valve product may contribute to SVD. However, the aforementioned clinical studies relied on echocardiographic measurements which were inadequate for comprehensive haemodynamic analyses.

Experimental studies have been performed to evaluate the performance of Magna Ease valve by visualizing its dynamics and the surrounding haemodynamics (Wendt et al., 2015; Tasca et al., 2022; Stephan et al., 2024; Raghav et al., 2016; Marx et al., 2018). Raghav et al. showed that the valve maintains an optimal EOA and regurgitant fraction even after undergoing durability tests simulating one billion cycles, equivalent to approximately 25 years of use (Raghav et al., 2016). Stephan et al. captured the complex flow pattern around the valve, including the formation of helixes and vortices, and wall shear stress along the aortic root by 4D flow magnetic resonance imaging (MRI) and vector flow Doppler ultrasound (Stephan et al., 2024).

However, *in vitro* experimental set-ups cannot fully capture the complexity of the real cardiovascular system, such as the varying tissue properties and anatomical variations. To address these limitations, computational fluid dynamics and FSI simulations have been employed (Kim et al., 2024; Abbasi et al., 2019) to provide more insights into the haemodynamics and biomechanics of BPAV, such as predicting wall shear stress (Sadipour, 2023) and mechanical stress (Kim et al., 2024; Abbasi et al., 2019; Sadipour, 2023) on the valve leaflets. These parameters are important due to their association with valve thrombosis (Sadipour, 2023) and durability of the valve product (Kim et al., 2024). However, previous FSI studies on surgical BPAVs adopted an idealized aortic root geometry or non-patient-specific flow conditions. Incorporating patient-specific factors will provide greater insights into the impact of individual anatomical and physiological variations on the performance of BPAV, leading to improved design and optimisation of valve products. There is currently no FSI model of the Magna Ease valve that takes into account patient-specific geometry and flow conditions. To address this gap, the current study focused on developing a fully coupled FSI model for patient-specific simulations of BPAV haemodynamics, and the Edwards Magna Ease valve was chosen as an example to demonstrate the developed workflow. Predicted flow features, including velocity streamlines, stroke volume, maximum jet velocity and peak systolic spatial mean velocity, were compared against those extracted from the corresponding *in vivo* 4D flow MRI data.

## 2 Materials and methods

### 2.1 Data acquisition and image processing

4D flow MRI and CT scans of the aorta were performed on a 66-year-old male patient (Height = 179 cm, Weight = 93 kg) implanted with a 25 mm Edwards Magna Ease valve. All scans were performed within 1 year after the valve replacement procedure. The MRI scan was performed with a Siemens Sola 1.5T at Norfolk and Norwich University Hospital (Norwich, United Kingdom). Images were acquired in the sagittal plane, at 30 time points within the cardiac cycle, and with a voxel size of 3.1 × 3.1 × 3.1 mm^3^. Velocity encoding parameters for the three velocity components (anterior-posterior, foot-head and right-left) were 4.0, 4.0 and 4.0 m/s, respectively. Data acquisition and handling were authorized by the National Research Ethics Service in the United Kingdom, with approval number 21/NE/0149.

**FIGURE 1:**
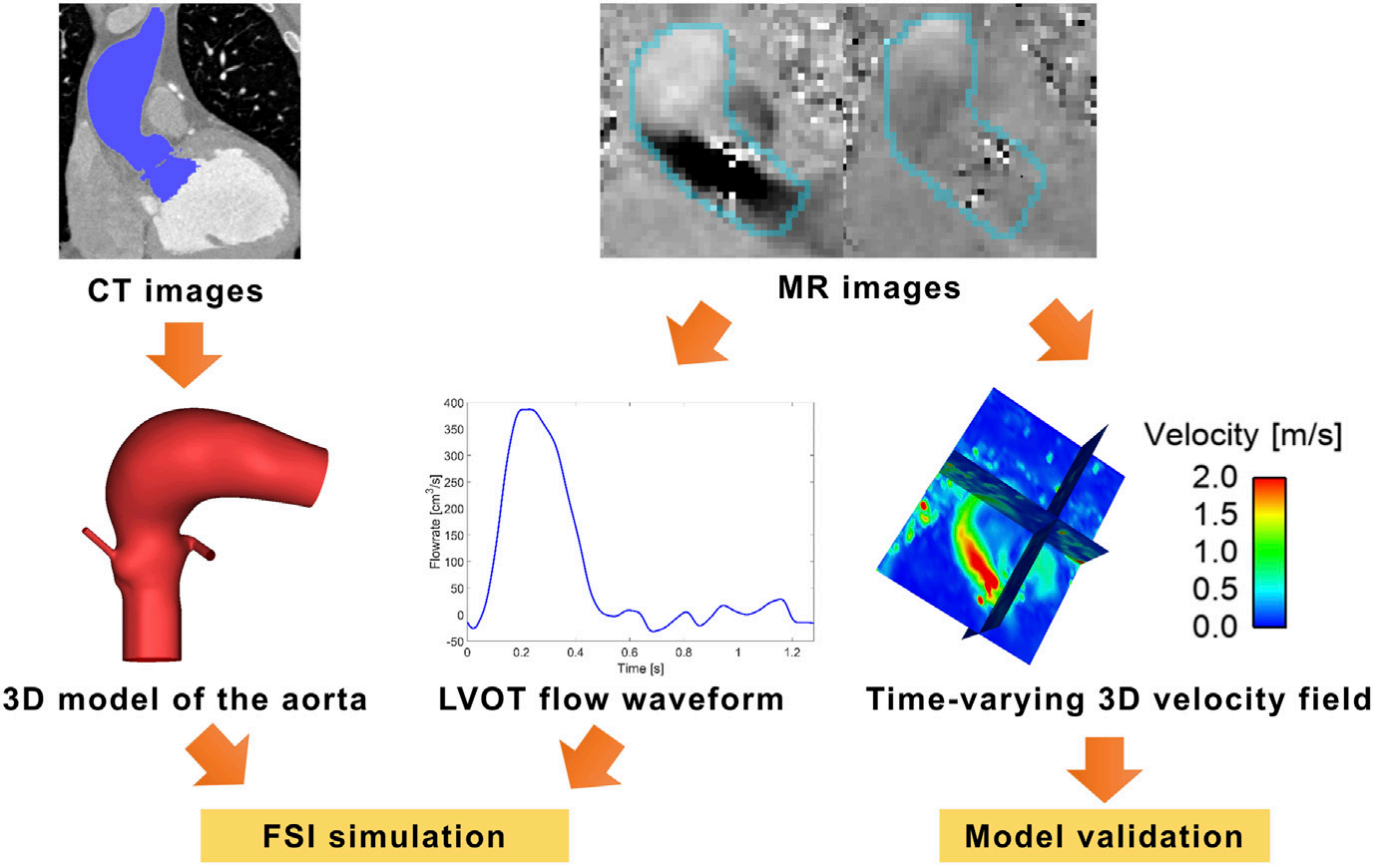
Reconstruction of the aortic root from CT scan and inlet flow waveform from MR images. LVOT–left ventricular outflow tract.

CT images were used to reconstruct a patient-specific model of the aortic root, using Materialise Mimics (v24.0, Materialise, Leuven, Belgium). The aortic root model included the distal end of the left ventricular outflow tract (LVOT), the ascending aorta and the proximal aortic arch, as well as the left and right coronary arteries. The raw 4D flow MR images were processed using an in-house Python code (Saitta, 2022), which generated 3D velocities in the aorta at each time point and saved these as a dataset block (.vtk format). The inlet flow waveform was then extracted in EnSight (Ansys Inc., United States) by applying the “flow” function to the LVOT plane from the dataset block, following our previously published methodology (Saitta et al., 2023; Zhu et al., 2025). The resulting aortic root geometry and inlet flow waveform are shown in Figure 1.

### 2.2 Modelling the valve product

**FIGURE 2:**
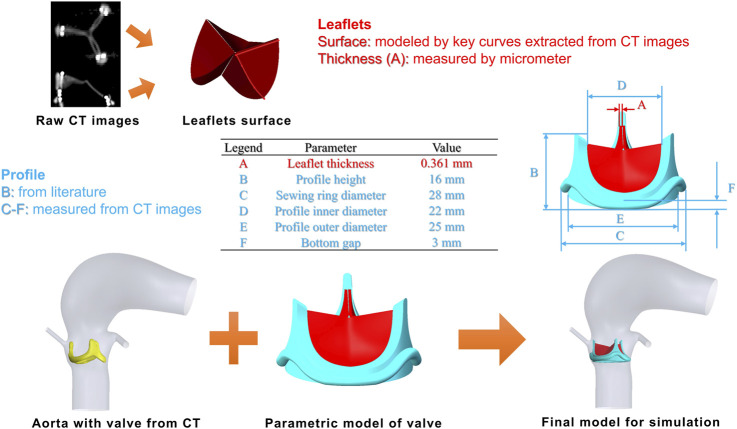
Parametric model of the 25 mm Edwards Magna Ease valve (top) and fitting of the valve product into a patient-specific aorta (bottom).

Building a geometric model of the Edwards Magna Ease valve product was accomplished in two steps: measuring key parameters from the valve and creating a parametric model. Geometric measurements were made with different tools. First, an unused 25 mm Magna Ease bioprosthesis was imaged *in vitro* using a clinical CT scanner at the Royal Brompton Hospital (Siemens Somatom Definition Flash, Siemens Medical Systems, Erlangen, Germany). The scan was performed with 4 cm z-axis coverage (0.6 mm collimation) centred over the valve prosthesis. As the spatial resolution was insufficient for direct reconstruction of leaflet surface and profile, the CT images were used to extract the free-edge curve and directrix curve of the valve leaflets (Figure 2) and to measure the profile parameters via Materialise Mimics. Then, the physical thickness of the valve leaflets was measured by a micrometre. Although the thickness varied in different areas of the leaflet, a uniform thickness was assumed, and the average value (0.361 mm) of the measurements made at 4 different locations was adopted.

Using the measurements and published data (Malaisrie et al., 2020; Becsek et al., 2020), a parametric model of the 25 mm Edwards Magna Ease valve was developed in SolidWorks (v2020, Dassault System, France). The valve leaflets were constructed via a sequential process: sweeping the free-edge curve along the directrix, followed by uniform thickness extrusion and trimming against the predefined inner surface profile. The integrated geometric model of the valve (leaflets and profile) was then translated and rotated to match the positions measured from the CT scan of the patient. To prevent unphysiological paravalvular leakage, the aorta was slightly smoothed in Meshmixer (Autodesk, United States) to ensure there were no gaps between the valve product and the aortic annulus. The reconstructed model including the valve is shown in Figure 2.

### 2.3 Blood properties and valve mechanical properties

Blood was modelled as an incompressible non-Newtonian fluid with a density of 1,060 kg/m^3^. Its shear-thinning viscous behaviour was described by the Bird-Carreau model where the blood viscosity μ can be written as:

$$\mu =\mu_{\infty }+\left(\mu_{0}-\mu_{\infty }\right){\left[1+{\left(\lambda \dot{\gamma }\right)}^{2}\right]}^{\frac{n-1}{2}}$$ (1)

where $\mu_{\infty }$ is the high-shear viscosity, $\mu_{0}$ is the low-shear viscosity, n is the power law index and λ is the time constant. In Equation 1, $\mu_{\infty }=0.0035\;Pa\cdot s$ , $\mu_{0}=0.056\;Pa\cdot s$ , n=0.3568 and λ=3.313 s (Cho and Kensey, 1991). A non-Newtonian model was adopted here because a recent study showed that the Newtonian assumption could underestimate shear stresses on the leaflets (Chen et al., 2022), even though it had a minor effect on the valve dynamics and transvalvular pressure (De Vita et al., 2015). Additionally, the flow was assumed to be laminar to avoid introducing additional complexities to the FSI simulation.

Valve leaflets were considered to be incompressible with a density of 1,100 kg/m^3^. Their material behaviour was described using an isotropic, hyperelastic second-order Ogden model (Ogden, 1972), and its strain-energy function is expressed in Equation 2:

$$\Psi =\sum_{i=1}^{N}\frac{2\mu_{i}}{\alpha_{i}^{2}}\left({{\bar{\lambda }}_{1}}^{\alpha_{i}}+{{\bar{\lambda }}_{2}}^{\alpha_{i}}+{{\bar{\lambda }}_{3}}^{\alpha_{i}}-3\right)$$ (2)

where $\mu_{i}$ and $\alpha_{i}$ are material constants, ${\bar{\lambda }}_{i}$ ( i=1,2,3 ) are the modified principal stretches. The parameters chosen for the leaflets were: N=2 , $\mu_{1}=19.58\;kPa$ , $\alpha_{1}$ = 67.74, $\mu_{2}=260.56\;kPa$ , and $\alpha_{2}$ = 27.47 (Mao et al., 2016), which were determined by fitting to stress-strain curves obtained from biaxial mechanical testing on 5 fresh glutaraldehyde-treated bovine pericardium samples (Sun, 2003). These parameters were adopted because the leaflets of Edwards Magna Ease valve were made of bovine pericardium. A Rayleigh damping coefficient of α = 200 s^−1^ was adopted for the leaflet material to consider the viscous damping effect. The valve profile and aortic wall were treated as rigid bodies. Previous comparative studies (Hsu et al., 2014; Marom et al., 2012) showed that accounting for arterial wall compliance could help dampen the oscillations during the valve closing phase, but it had minor influence on key features of valve dynamics and the surrounding haemodynamics.

### 2.4 Geometric discretization

The fluid domain (the aortic lumen) was discretised using FlowVision (CAPVIDIA, Leuven, Belgium) with the sub-grid geometry resolution (SGGR) method (Aksenov et al., 1998; Aksenov et al., 2004). The discretisation involved two steps: defining an initial Cartesian grid with a characteristic dimension ( $l_{0}$ ), followed by local refinement in regions near the aortic wall and leaflets. The fluid mesh sensitivity test was performed on the whole aorta with a static, fully opened valve, and the same boundary conditions as in the FSI simulation. Average velocities at three planes distal to the valve and shear stresses on the leaflets were calculated and compared between consecutive meshes. The results (Table 1) showed that a global mesh size of $l_{0}$ = 0.7 mm (M3) with a four-layer local refinement near the leaflets (local mesh size = 1/4×global mesh size) was adequate for good convergence in downstream velocity (<3% difference) and shear stress on the valve leaflets (<4% difference). This result was used to set up the computational FSI models, and the final fluid mesh was 124 × 301 × 178.

**TABLE 1:** Fluid mesh sensitivity test results for the aortic root model with a 25 mm Edwards Magna Ease valve (DOWN1, DOWN2 and DOWN3 represent 3 downstream planes distal to the valve; NCL, RCL and LCL stand for non-coronary, right coronary and left coronary leaflet, respectively).

Name	Grid number	Averaged velocity DOWN1 (m/s)	Averaged velocity DOWN2 (m/s)	Averaged velocity DOWN3 (m/s)	Averaged WSS NCL (Pa)	Averaged WSS RCL (Pa)	Averaged WSS LCL (Pa)
M1	461,455	0.281	0.276	0.283	2.891	3.136	3.064
M2	799,682	0.280	0.264	0.274	2.940	3.203	3.318
M3	1,129,200	0.276	0.266	0.278	3.095	3.226	3.443
M4	1,675,216	0.283	0.266	0.277	3.184	3.356	3.448
Difference (%)	M1-M2	0.303	4.547	3.195	1.649	2.093	7.650
	M2-M3	1.633	0.635	1.359	4.996	0.727	3.627
	M3-M4	2.596	0.017	0.521	2.815	3.879	0.127

The solid domain consisted of three leaflets and the valve profile. The three leaflets were discretised with C3D8R hexahedral elements in Abaqus (SIMULIA, Dassault System, France). The reasons for choosing solid instead of shell elements are twofold: first, using shell elements can result in self-intersection and negative volumes which would cause the simulation to fail; second, defining the two sides of the leaflets as boundary surfaces is crucial for accurately transferring information between the structural and fluid solvers. Solid elements provide a more robust framework for these boundary definitions, ensuring a stable and accurate fluid-structure interaction. The solid mesh sensitivity test was performed in decoupled finite element simulations with Abaqus, where the leaflets (closed initially) were pressurised by applying a uniform loading of 10 mmHg on the ventricular side. The results (Table 2) showed that a global solid mesh size of 0.25 mm and four elements in the thickness direction (M2) were sufficient to achieve <3% difference in geometric orifice area and maximum deformation compared to the finest mesh, so M2 was adopted in the FSI simulation where the final solid mesh was ∼47,000. The valve profile was assumed as a rigid body and discretised with R3D3 tetrahedral elements. Meshing the valve profile was necessary to allow suitable boundary conditions to be applied, and a global solid mesh size of 0.2 mm was adopted for this purpose.

**TABLE 2:** Solid mesh sensitivity test results for a 25 mm Edwards Magna Ease valve.

Name	Mesh number	GOA (cm^2^)	Max deformation (mm)
M1	36,660	2.175	10.010
M2	46,512	2.209	10.030
M3	82,356	2.250	9.961
M4	144444	2.252	9.814
Difference (%)	M1	3.420	1.997
	M2	1.910	2.201
	M3	0.053	1.498

### 2.5 Boundary conditions and co-simulation set-ups

**FIGURE 3:**
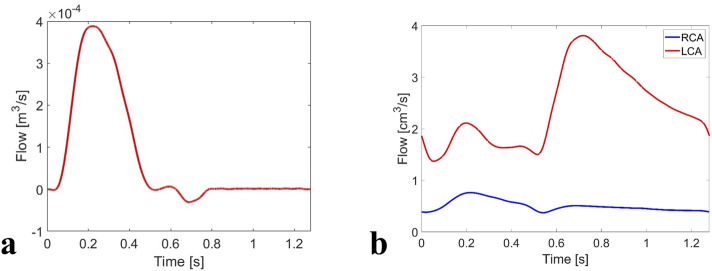
Boundary conditions of the FSI simulation: **(a)** flow waveform applied at the LVOT inlet, and **(b)** flow waveforms applied at the coronary outlets.

In the fluid domain, the flow waveform derived from 4D flow MRI (Figure 1), assuming zero diastolic flow (Figure 3A), was applied at the inlet located in the LVOT along with a flat velocity profile. The descending aorta outlet was extended by 125.4 mm, approximately five times the local diameter, and a three-element Windkessel (3EWK) model was applied at the outlet. For this case, a mean pressure of $\bar{P}$ = 93.33 mmHg was used, which was estimated by assuming aortic blood pressure of 120/80 mmHg. It also accounted for the pressure loss (2.9 Pa for this case) linked to the artificial outlet extension, which was estimated by the Hagen-Poiseuille equation. The mean inlet flow $\bar{Q}$ was calculated from the inlet flow. Using the estimation method proposed in (Pirola et al., 2017), the 3EWK parameters were calculated and their values are given in Table 3.

**TABLE 3:** 3EWK parameters for the model outlet. $R_{p}$ : proximal resistance, $R_{d}$ : distal resistance, C : compliance.

$R_{p}$ (kg/m^4^s)	$R_{d}$ (kg/m^4^s)	C (m^4^s^2^/kg)
1.004 × 10^7^	1.574 × 10^8^	1.069 × 10^−8^

At the coronary outlets, flow waveforms corresponding to a healthy volunteer (Kim et al., 2010) were scaled to fit the current case in two steps. First, the left coronary artery (LCA) and right coronary artery (RCA) waveforms were scaled along the time-axis to match the systolic and diastolic phase extracted from the LVOT flow waveform; second, the height of the resulting waveforms was scaled to ensure the time-averaged flow was 3.1% and 0.65% of the LVOT flow for the LCA and RCA (Kandail et al., 2018), respectively. The coronary flow distributions were not patient-specific, but based on haemodynamic data from healthy adults (Kim et al., 2010). Consequently, they represent a physiologically reasonable approximation for population-level simulations under baseline conditions. The modified flow waveforms (Figure 3B) were applied at the coronary outlets.

In the structural domain, a tie constraint was applied between the valve leaflets and profile, and the profile was fixed in all degrees of freedom. A contact model was defined between the three leaflets, which was based on a hard normal pressure-overclosure formulation with a tangential friction coefficient of 0.1.

A two-way FSI coupling was defined in Abaqus Explicit 2019 (SIMULIA, Dassault System, France) and FlowVision (CAPVIDIA, Leuven, Belgium) at an exchange step of 0.1 ms. An implicit time integration scheme was used in FlowVision with a constant time-step equal to the exchange step (0.1 ms), while the minimum explicit time-step used in Abaqus was approximately 1 × 10^−7^ s. At every exchange step, valve deformation was solved in Abaqus Explicit solves using the concentrated forces obtained from a previous step as a loading condition. The deformed geometry was then transferred to FlowVision to calculate velocity and pressure fields and concentrated forces on the valve. These forces were sent back to Abaqus Explicit to be used as the new load for the next exchange step. This cycle repeated until the end of the simulation which included three cardiac cycles to reach a periodic solution. The FSI simulation was performed on an Intel(R) Xeon(R) Silver 4114 CPU @ 2.20 GHz 2.19 GHz (two processors), 128 GB RAM workstation, and it took approximately 19 days. Results obtained from the third cycle were extracted for subsequent analysis.

### 2.6 Analysis of results

Computational results for flow characteristics and valve motion were analysed at representative time points throughout the third cardiac cycle. All postprocessing was conducted in FlowVision Viewer, Abaqus Visualization module, Ensight, Paraview and Matlab.

The performance of the valve was evaluated by calculating the geometric orifice area (GOA), effective orifice area (EOA), aortic valve area (AVA), transvalvular pressure gradient (TPG) and mean pressure gradient (MPG). GOA refers to the anatomical area of an opening valve, and it can be calculated from a 2D projection of the AV leaflets’ free edge on the aortic root cross-sectional area (Votta et al., 2017). EOA describes how effectively the valve opens during the forward flow phase and is computed from the principle of energy conservation defined in Equation 3 (International Organization for Standardization, 2021):

$$\text{EOA}=\frac{Q_{\text{RMS}}}{51.6\sqrt{∆\bar{P}/\rho_{f}}}$$ (3)

where $∆\bar{P}$ refers to the mean pressure difference during the positive differential pressure period (mmHg), $\rho_{f}$ is the fluid density (g/cm^3^), and $Q_{RMS}$ is the root mean square forward flow (mL/s) during the positive differential pressure period.

The orifice area can also be evaluated based on the continuity equation by dividing stroke volume (SV) by the maximum velocity integral across the valve, the latter can be derived from echocardiographic images. This is called AVA, which is defined in Equation 4 (Baumgartner et al., 2009):

$$\text{AVA}=\frac{\text{SV}}{{VTI}_{\text{AV}}}$$ (4)

where ${VTI}_{AV}$ indicates the maximum velocity time integral across the aortic valve over the ejection period.

The mean transvalvular pressure gradient (TPG) during the systolic phase has been used to quantify the potential energy loss as blood flows through the aortic valve (Cai et al., 2021). It is defined in Equation 5:

$$\text{TPG}=\frac{\int_{t_{bs}}^{t_{es}}\left(P_{n\text{LV}}-P_{n\text{AO}}\right)dt}{t_{es}-t_{bs}}$$ (5)

where $P_{n\text{LV}}$ and $P_{n\text{AO}}$ are the pressures near the leaflets at the left ventricular and aortic sides (measured at 20 mm downstream and upstream the valve in this case), and $t_{bs}$ and $t_{es}$ are the beginning and end of systole.

In clinical practice, another similar parameter, namely, mean pressure gradient (MPG), which can be derived from echocardiographic images, has been widely used. It is based on the Bernoulli equation by neglecting viscous losses and acceleration effects, and is defined in Equation 6 (Baumgartner et al., 2009):

$$\text{MPG}=\frac{\sum_{i=1}^{N}4v_{\text{AV}\max}^{2}}{N_{tp}}$$ (6)

where $v_{\text{AVmax}}$ is the maximum velocity across the valve, $N_{tp}$ is the number of time points measured when the valve is open. Here, the units of velocity and pressure are m/s and mmHg, respectively.

Wall shear stress (WSS) is an important haemodynamic parameter; its magnitude and directional variation have been correlated with aorto-pathology and valve degeneration (Salmasi et al., 2023; Hanna et al., 2022). Both peak-systolic (PWSS) and time-averaged wall shear stress (TAWSS) on the aortic wall and leaflets were analysed. Other time-averaged metrics, including oscillatory shear index (OSI) and relative residence time (RRT), representing directional variation, and the relative time blood spends on the wall respectively, were also evaluated and compared among the three leaflets. Their definitions are given in Equations 7-9:

$$\text{TAWSS}=\frac{\int_{0}^{T}\left|\tau \right|\text{dt}}{T}$$ (7)

$$\text{OSI}=\frac{1}{2}\left(1-\frac{\left|\int_{0}^{T}\tau \text{dt}\right|}{\int_{0}^{T}\left|\tau \right|\text{dt}}\right)$$ (8)

$$\text{RRT}=\frac{1}{\left(1-2\times \text{OSI}\right)\times \text{TAWSS}}$$ (9)

where τ represents the WSS vector at a particular time, and T is the cardiac cycle.

## 3 Results

### 3.1 Flow patterns and comparison with 4D flow MRI

**FIGURE 4:**
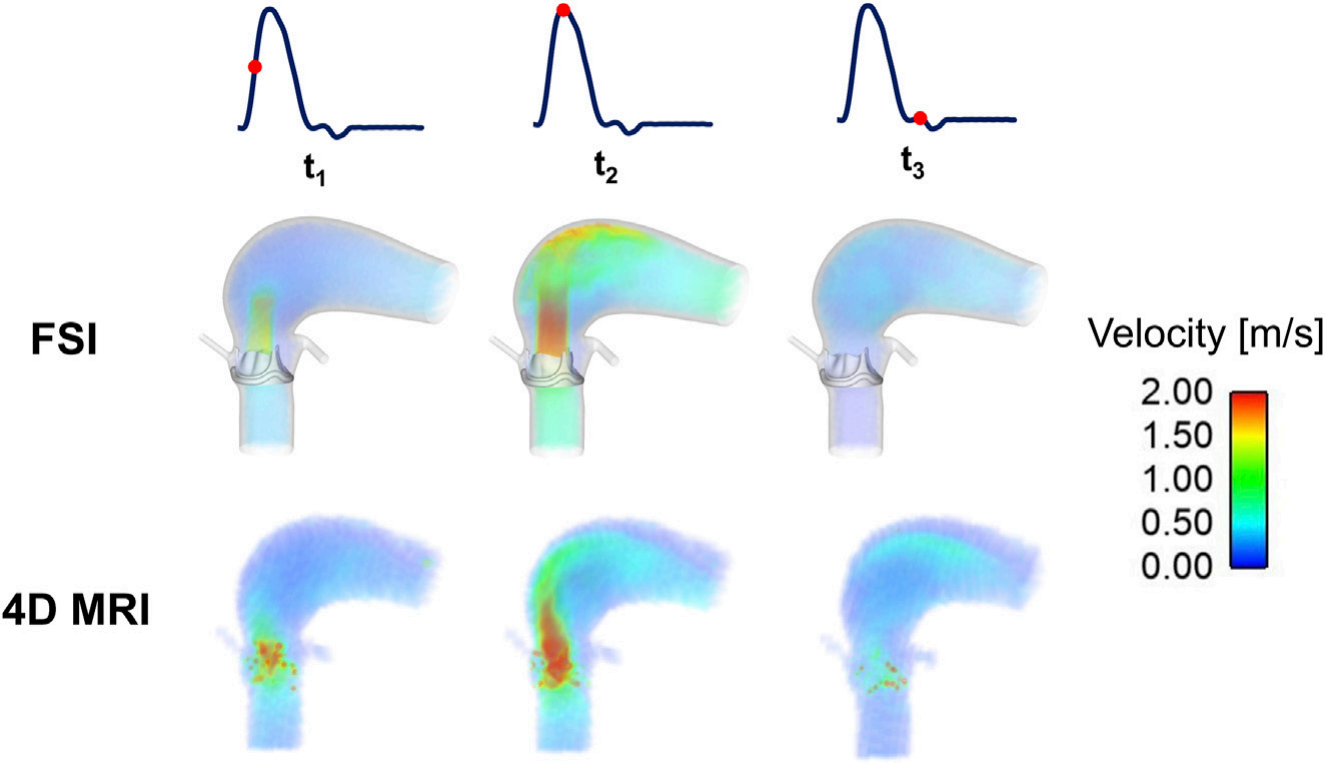
Velocity 3D contours at three representative time points (t_1_: acceleration phase, t_2_: peak-systole, and t_3_: deceleration phase) for the FSI and 4D flow MRI results.

Figure 4 shows velocity 3D volume rendering contours obtained from the FSI simulation, alongside the corresponding 4D flow MRI measurements. The time-varying flow pattern from the FSI simulation is qualitatively similar to that observed in the healthy case (Yin et al., 2024), and the MRI data exhibit a spatial flow pattern comparable to that of the FSI simulation. However, there are quantitative differences. At peak systole, the FSI results show higher velocities in the aortic arch. It should be noted that spurious velocities (displayed as red dots) are observed around the valve device in the 4D flow MR images. These are likely artifacts caused by the interaction of the cobalt-chromium alloy with the magnetic field during the MRI scan, leading to signal distortion in the imaging data.

**FIGURE 5:**
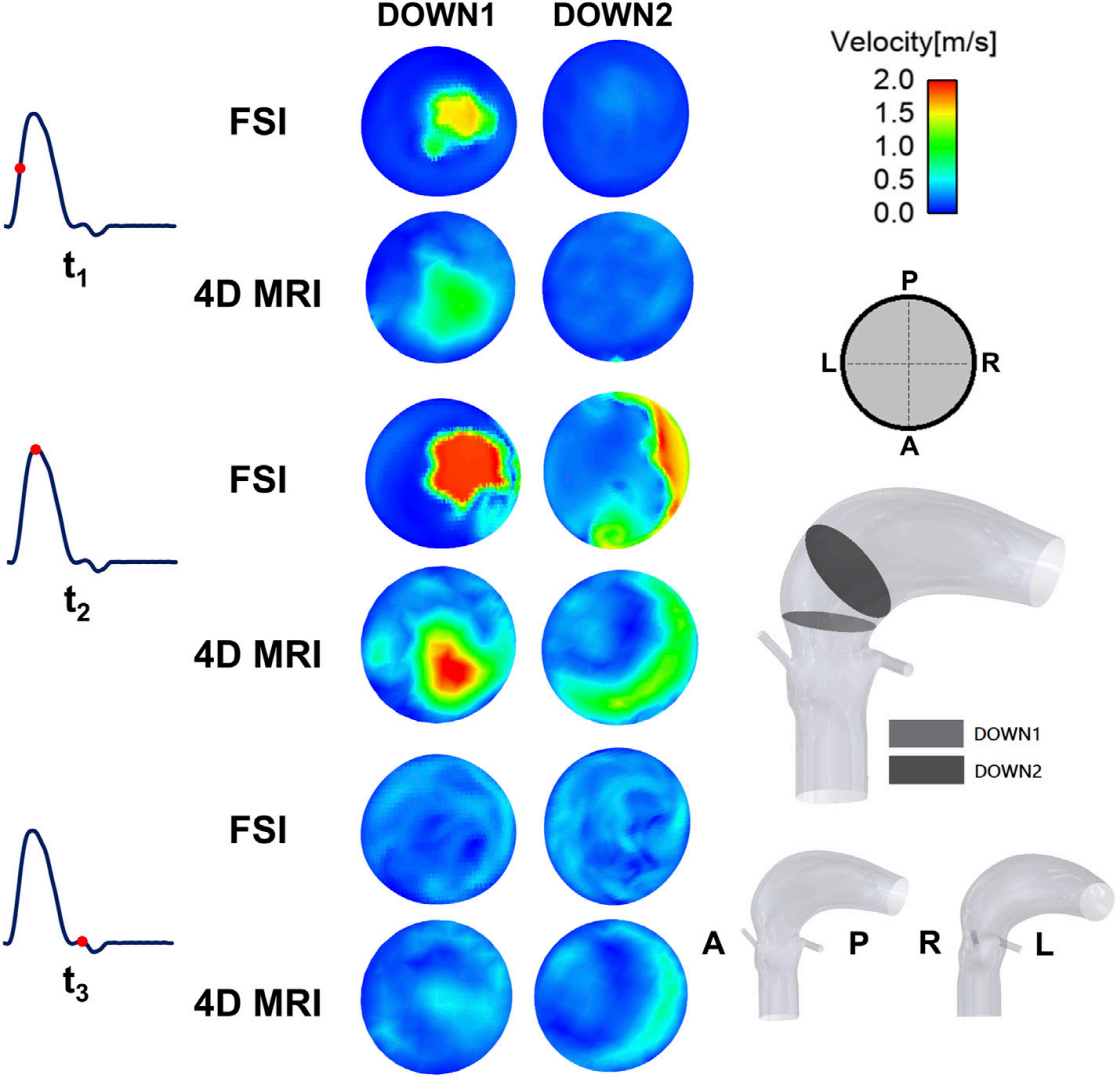
Velocity contours at three representative time points (t_1_: acceleration phase, t_2_: peak-systole, and t_3_: deceleration phase) for the FSI and 4D flow MRI results.

Figure 5 shows comparisons of velocity contours at two cross-sections downstream of the valve. At the plane closer to the valve (DOWN1), both the FSI simulation and MRI data display a distinct jet flow at the middle right location of the cross-section at t_1_ and t_2_, but with different magnitudes. Further distal from the valve (DOWN2), both FSI and MRI results show a crescent-like contour covering the right-anterior side.

A quantitative comparison of key parameters was made between the simulation results and 4D flow MRI data (Table 4). Here, stroke volume was calculated by integrating instantaneous flow rate over time during systole. The jet velocity and peak systolic spatial mean velocity were measured at the DOWN1 plane defined in Figure 5. Compared to the MRI measurements, the FSI simulation predicted a slightly lower SV (−0.43%), higher maximum jet velocity (+5.77%) and a lower peak systolic spatial mean velocity (−4.69%) (Table 4).

**TABLE 4:** Quantitative comparison of key parameters derived from FSI simulation results and 4D-flow MRI. The jet velocity and peak systole spatial mean velocity were measured at a transverse section 20 mm above the sinus plane. SV, stroke volume.

Parameter	4D-flow MRI	FSI	Difference (%)
SV (mL)	99.91	99.48	−0.43%
Max Jet Velocity (m/s)	2.08	2.20	5.77%
Peak Systolic Spatial Mean Velocity (m/s)	0.64	0.61	−4.69%

**FIGURE 6:**
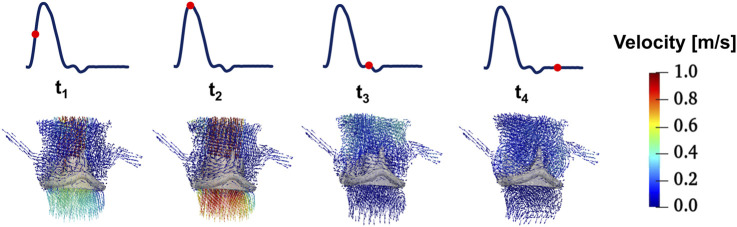
3D velocity vectors in the area surrounding the valve throughout the cardiac cycle (t_1_: acceleration phase, t_2_: peak-systole, t_3_: deceleration phase, and t_4_: mid diastole phase).

Figure 6 shows 3D velocity vectors in the area surrounding the valve throughout the cardiac cycle. At all timepoints, low-velocity recirculation zones are consistently observed between the valve leaflets and sinus. These regions exhibit reduced shear rates, leading to locally elevated blood viscosity due to its shear-thinning behaviour (Equation 1). It suggests that sustained viscous effects in these zones may stabilize flow patterns, potentially mitigating flow stagnation risks associated with thrombus formation.

### 3.2 Valve dynamics

**FIGURE 7:**
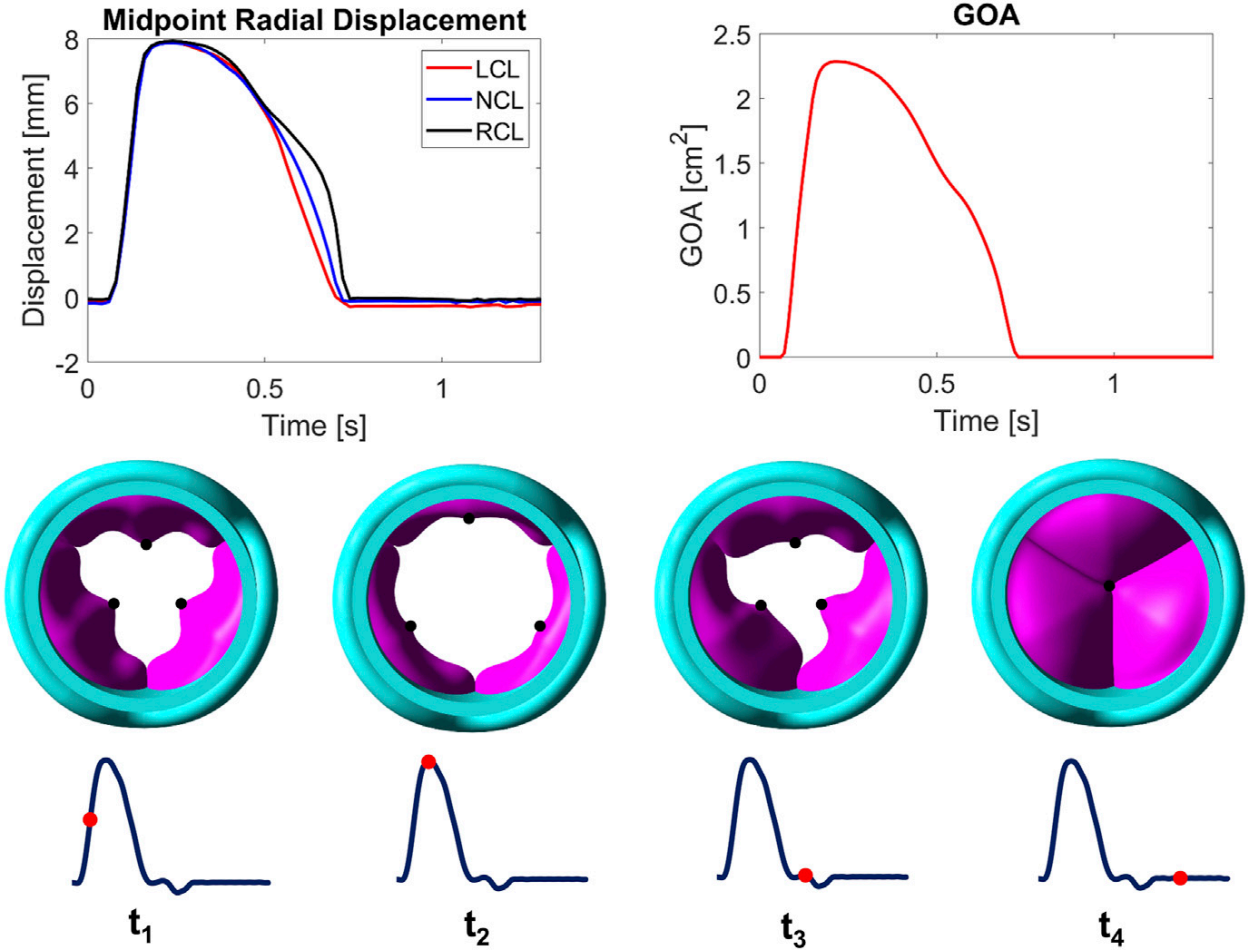
Midpoint radial displacements for LCL, NCL and RCL (top left) and geometric orifice area (GOA, top right) throughout the cardiac cycle. Valve leaflet configurations (viewed from the LVOT, vertical to the centreline) at selected time points during a cycle (bottom). LCL, NCL and RCL refer to the left coronary, non-coronary, and right coronary leaflet, respectively.

Figure 7 presents dynamic parameters of the valve throughout the cardiac cycle and valve leaflet configurations at four distinct time points. The midpoint radial displacements for the three leaflets with respect to their initial unloaded positions are almost identical before the maximum value reaching 7.88 mm, 7.86 mm and 7.93 mm for the LCL, NCL and RCL, respectively. During late systole, the variation in slope indicates that the three valve leaflets have different closing speeds. During diastole, the LCL midpoint displacement is smaller than that of the other two leaflets and less than zero. Similar to what was observed from the healthy valve (Yin et al., 2024), the time-varying GOA also reaches a maximum (2.28 cm^2^) at peak systole, before reducing to zero when the valve is closed. The valve orifice shape is triangular when the valve is opening (t_1_) and closing (t_3_), and more circular when the valve is fully open (t_2_).

### 3.3 WSS on the aorta and leaflets

**FIGURE 8:**
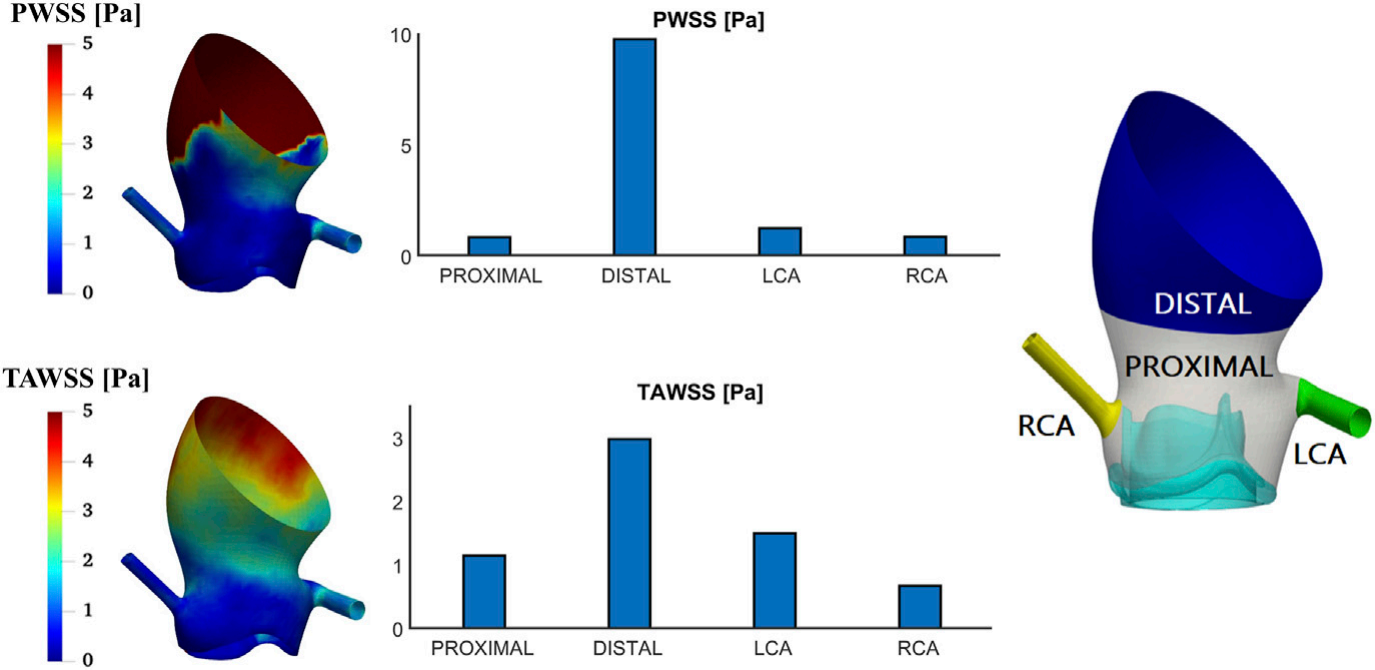
Wall shear stress at peak systole (PWSS) and time-averaged wall shear stress (TAWSS) contours on the aortic root and spatially averaged values in different areas of interest.

Figure 8 shows the PWSS and TAWSS on the aortic root. Among all areas, WSS is highest in the DISTAL segment where the maximum PWSS and TAWSS reach 9.77 Pa and 2.99 Pa, respectively. The lowest WSS occurs in different regions for PWSS and TAWSS, with values of 0.84 Pa in the PROXIMAL segment and 0.67 Pa in the right coronary artery, respectively. The spatial variations of PWSS and TAWSS along the aorta follow a similar pattern.

**FIGURE 9:**
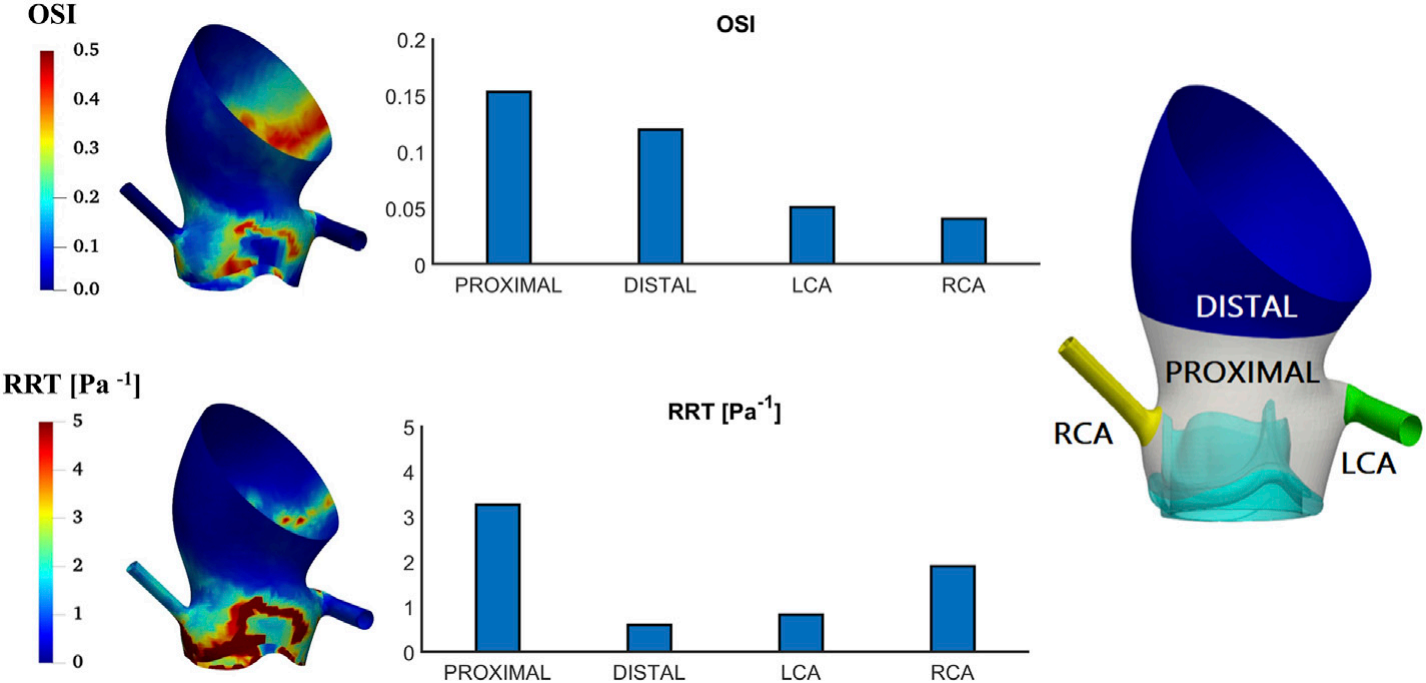
Oscillatory shear index (OSI) and relative residence time (RRT) contours on the aortic root and spatially averaged values in different areas of interest.

Figure 9 shows OSI and RRT patterns on the aortic root. Among all areas, the highest values are observed in the PROXIMAL area where the maximum OSI and RRT reach 0.15 and 3.27 Pa^−1^, respectively. The lowest values occur in different regions for OSI and RRT, with OSI of 0.04 in the right coronary artery and RRT of 0.60 Pa^−1^ in the DISTAL area. The spatial variations of OSI and RRT along the aorta follow a similar pattern.

**FIGURE 10:**
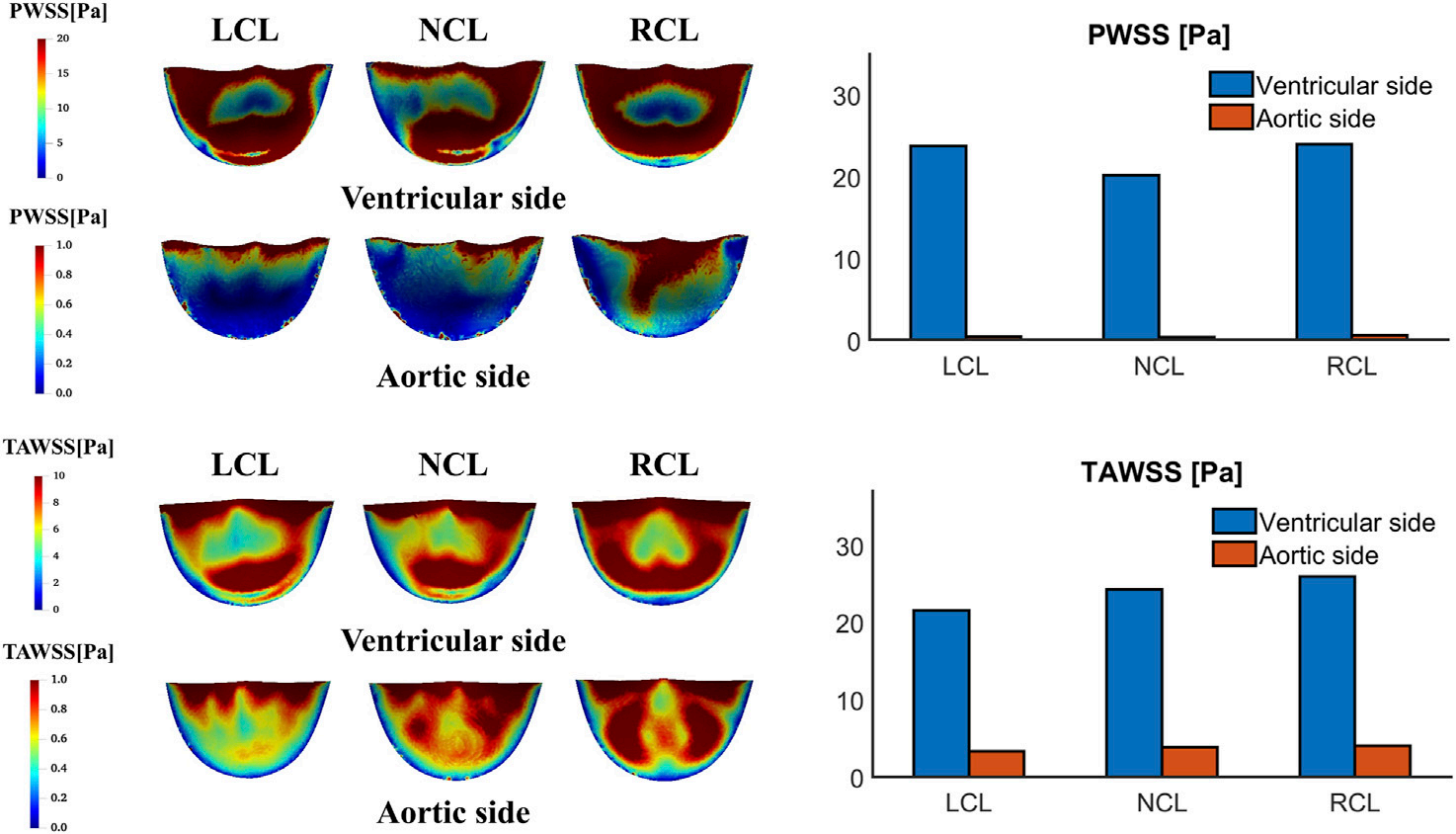
Wall shear stress at peak systole (PWSS) and time-averaged wall shear stress (TAWSS) contours on the ventricular and aortic surfaces of the leaflets and their spatially averaged values on each leaflet. LCL, NCL and RCL refer to the left coronary, non-coronary, and right coronary leaflet, respectively.

Figure 10 shows the PWSS and TAWSS contours on the two sides of the leaflets. Both PWSS and TAWSS are higher on the ventricular side than the aortic side. On the ventricular side, the NCL has the smallest PWSS (20.20 Pa compared to 23.74 Pa and 23.95 Pa for the LCL and RCL, respectively) and the LCL has the smallest TAWSS (21.58 Pa compared to 24.32 Pa and 25.94 Pa for the NCL and RCL, respectively). On the aortic side, the RCL has the largest PWSS (0.63 Pa compared to 0.46 Pa and 0.39 Pa for the LCL and NCL, respectively) and the largest TAWSS (4.11 Pa compared to 3.36 Pa and 3.87 Pa for the LCL and NCL, respectively). High WSS areas are mostly located near the top edges of the leaflets on both sides and the middle to bottom region on the ventricular side.

**FIGURE 11:**
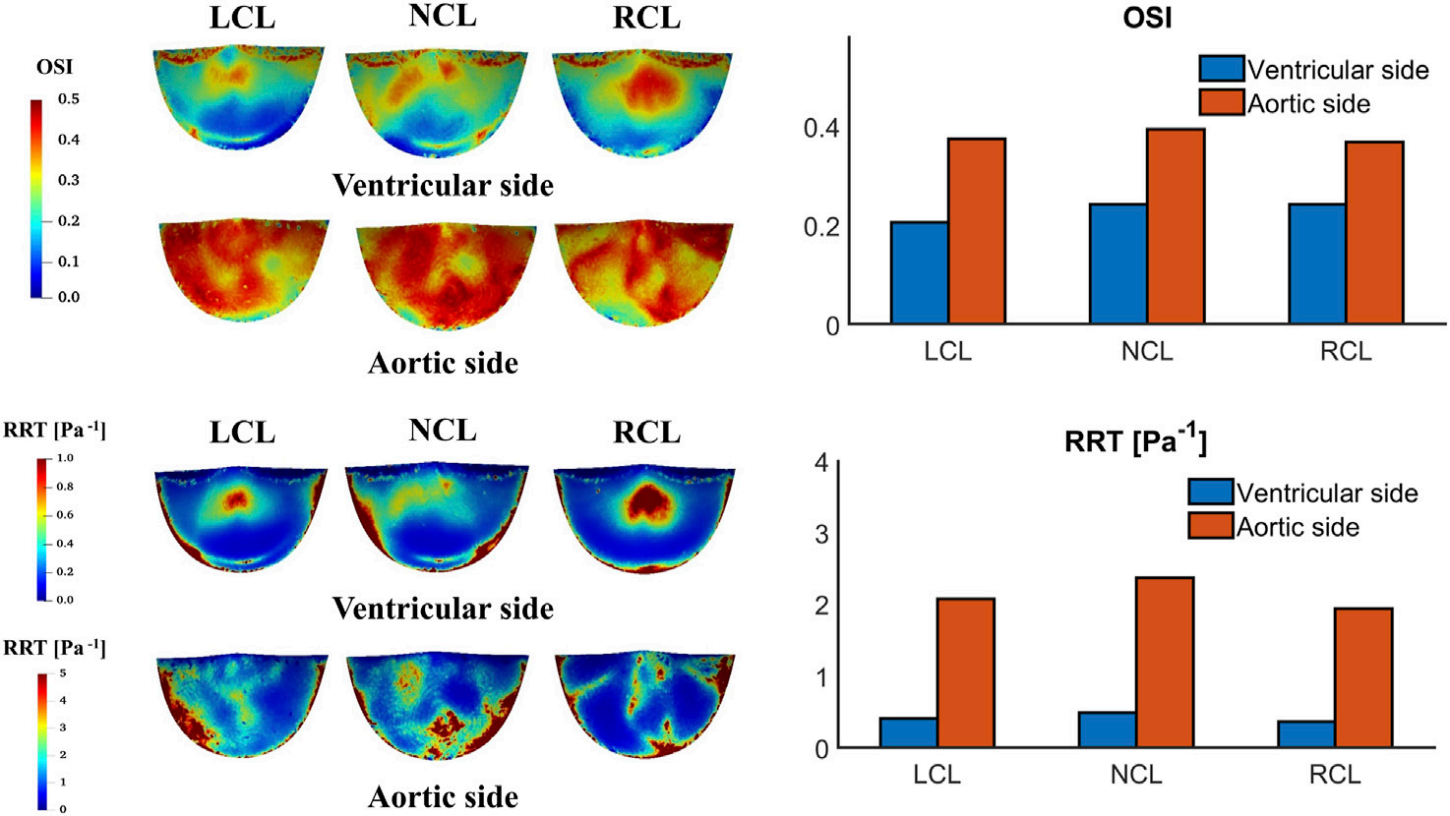
Oscillatory shear index (OSI) and relative residence time (RRT) contours on the ventricular and aortic surfaces of the leaflets and their spatially averaged values on each leaflet.

Figure 11 shows the OSI and RRT contours on the two sides of the leaflets. Both OSI and RRT are higher on the aortic side than the ventricular side. On the ventricular side, the LCL has the smallest OSI (0.21 compared to 0.24 and 0.24 for the NCL and RCL, respectively) and the RCL has the smallest RRT (0.37 Pa^−1^ compared to 0.41 Pa^−1^ and 0.49 Pa^−1^ for the LCL and NCL, respectively). On the aortic side, the NCL has the largest OSI (0.39 compared to 0.38 and 0.37 for the LCL and RCL, respectively) and the largest RRT (2.37 Pa^−1^ compared to 2.08 Pa^−1^ and 1.94 Pa^−1^ for the LCL and RCL, respectively). The spatial distributions of OSI and RRT are similar, with the highest magnitudes observed along the attachment edge on both sides and in the central region on the ventricular side.

## 4 Discussion

In this study, an improved FSI simulation workflow has been developed and applied to an Edwards Magna Ease valve in a patient-specific setting. The workflow includes the creation of a geometric model by combining the aortic root reconstructed from CT images and the valve created based on available measurements made on the device. Two-way fully coupled FSI simulations have been performed under patient-specific flow conditions. Simulation results are also compared with 4D flow MRI in an initial attempt to validate the model.

### 4.1 Comparison of FSI simulation results with 4D flow MRI measurements

In this study, the evaluation of the Magna Ease valve demonstrated a good agreement between the FSI simulation and 4D flow MRI in SV, maximum jet velocity, peak systolic mean velocity (Table 5), and in overall flow pattern (Figure 4). By applying the 4D flow MRI-derived flow waveform at the model inlet, it was possible to ensure that the measured *in vivo* LVOT flow rate was faithfully reproduced in the FSI simulation. This was an improvement over our previous study where a scaled pressure waveform was used as the inlet boundary condition (Yin et al., 2024), which resulted in a mismatch between the computed and 4D flow MRI measured flow waveform at the LVOT inlet. However, differences in the magnitude of jet flow at the selected cross-sections (Figure 5) still exist. These discrepancies can be partially attributed to inaccuracies inherent to 4D flow imaging. For example, a single and high VENC (4.0 m/s) setting was selected to enhance scanning efficiency and prevent velocity aliasing, however, this may reduce accuracy in low-velocity regions. Additionally, the MRI data had limited spatial resolution (voxel size 3.1 × 3.1 × 3.1 mm^3^) and temporal resolution (30 time points over a cardiac cycle). From the modelling point of view, the shape and material properties of the leaflets were based on available measurements and information in the literature, which may not represent the exact geometry and behaviour of the implanted valve. In addition, the aortic wall compliance and device profile were neglected in the FSI simulation. All these may affect the predicted valve opening area, and in turn jet velocities.

### 4.2 Evaluation of the valve’s haemodynamic performance and comparison to literature

The haemodynamic performance of different valve products has been evaluated by researchers using a variety of techniques, including *in vivo* imaging, *in vitro* experiments, and FSI simulations. Previous evaluations of the Magna Ease valve have primarily relied on echocardiography data across large cohorts of patients. Some experimental studies have also been performed on testing the valve’s performance in idealized aortic root models. However, FSI simulations of the Magna Ease valve under patient-specific conditions have not been reported in the literature.

**FIGURE 12:**
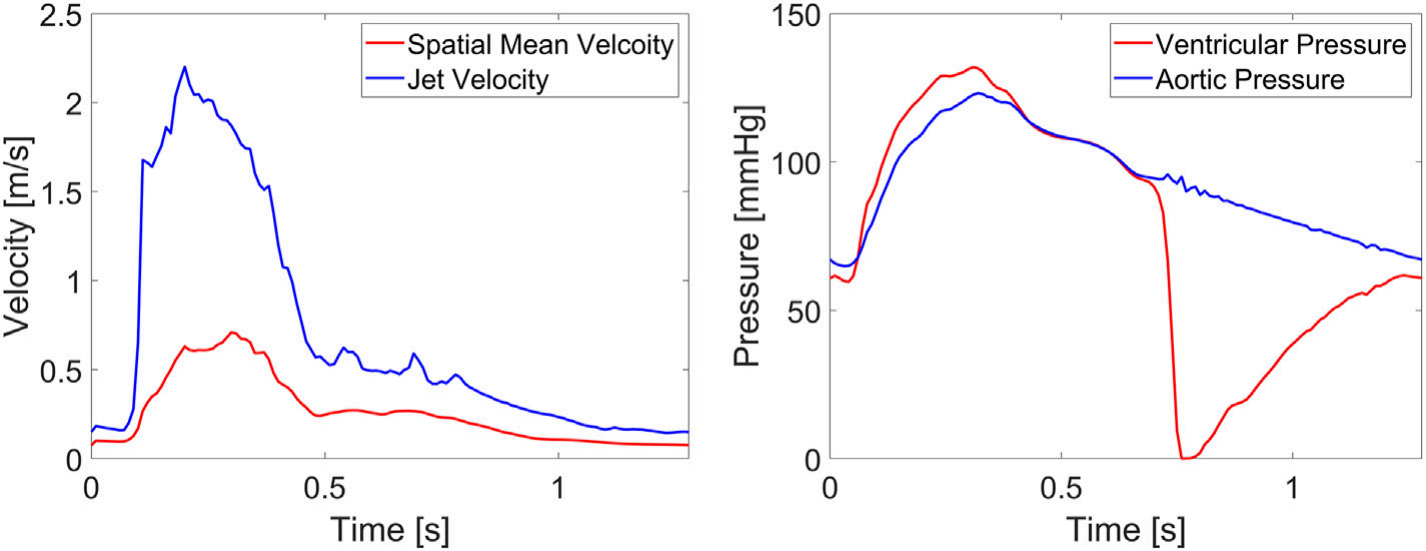
Variations of velocity and pressure over time. The spatial mean velocity, jet velocity and aortic pressure were measured at a transverse section 20 mm above the sinus plane, ventricular pressure was measured at a transverse section 20 mm below the sinus plane.

Our FSI simulation results provide key haemodynamic functional parameters for valve performance, which can be compared with those obtained *in vivo*. The maximum jet velocity from the simulation is 2.20 m/s, which falls within the range of 1.84–2.62 m/s obtained from echocardiography measurements in patients implanted with a 25 mm Magna Ease valve (Shala and Niclauss, 2020). The EOA from the simulation is 1.99 cm^2^, exceeding the minimum aortic valve EOA requirement of 1.45 cm^2^ for a 25 mm valve as specified by ISO 5840–2. The AVA was calculated by dividing SV by the maximum velocity integral across the valve (see velocity data in Figure 12) and the result from the simulation is 1.49 cm^2^, which is within the range of 1.41–2.21 cm^2^ obtained from echocardiography measurements in patients implanted with a 25 mm Magna Ease valve (Mayr et al., 2021). Values for TPG and MPG from the FSI simulation are 5.82 mmHg and 8.10 mmHg, respectively. TPG was derived directly from the simulated ventricular and aortic pressure data (Figure 12), which cannot be directly compared to *in vivo* measurements due to the lack of non-invasive methods for pressure measurement. MPG is within the range of 8–15 mmHg obtained from echocardiography measurements in patients implanted with a 25 mm Magna Ease valve (Shala and Niclauss, 2020). For the patient included in this study, MPG calculated from the corresponding echocardiography measurements is 15 mmHg, which is nearly twice the value obtained from the simulation. Plausible reasons for the discrepancies can be explained as follows. First, MPG (defined in Equation 6) is strongly dependent on both the maximum velocity across the valve and the duration of systole. Notably, any error in estimating maximum velocity is amplified by the square term in this equation, making accurate velocity measurement particularly critical. Consequently, the large underestimation of MPG in the FSI simulation compared to echocardiographic measurement is primarily attributed to an underestimated maximum velocity, which results from an overestimated valve opening area (Figure 5), given that the flow rate remains identical between the simulation and measurement. Furthermore, Equation 6 is based on the Bernoulli equation, which introduces a substantial assumption that cannot fully capture the complexity of flow around the valve.

In addition to the above parameters, the FSI simulation offers a more detailed analysis of the local haemodynamics of the Magna Ease valve, providing insights that conventional techniques cannot deliver. Figures 4, 5 show the 3D spatial and 2D cross-sectional distributions of the blood velocity with high spatial (approximately 0.7 × 0.7 × 0.7 mm^3^) and temporal resolution (every 0.1 ms), exceeding that of 4D flow MRI and other conventional imaging techniques. The experimental studies on the Magna Ease valve by Marx et al. (2018) and Marx et al. (2020) provided high-resolution (900 μs and 20–50 μm) 2D axial velocity visualization using high-speed cameras and particle image velocimetry systems. These studies revealed a pronounced central jet surrounded by regions of lower velocity magnitude at peak systole, consistent with our observations (Figures 4, 5). Another study of the Magna Ease valve (Stephan et al., 2024) utilised 4D flow MRI in combination with sonographic imaging, achieving a temporal resolution similar to our 4D flow (25 timepoints per cardiac cycle) and a spatial resolution comparable to our FSI simulations (1.0 × 1.0 × 1.0 mm^3^). This study observed a centrally symmetric velocity distribution shifted toward the outer curvature, creating an asymmetric flow pattern at peak systole, also reflected in our velocity contours (Figures 4, 5).

Furthermore, it is possible to evaluate wall shear stress and its derivatives on the valve leaflets. WSS indicates the level of frictional stress exerted on the endothelial lining of the vessel wall or valve surface (Armour et al., 2022), and areas exposed to high WSS might be at high risk of endothelial damage, leading to aorta-pathology and valve degeneration (Hanna et al., 2022; Salmasi et al., 2023). Elevated OSI and RRT are associated with thrombus formation (Mutlu et al., 2023; Laha et al., 2024), which could be used to evaluate the risk of leaflet thrombosis. As shown in Figures 10, 11, asymmetric patterns are observed among the leaflets: RCL has the highest TAWSS on the ventricular side and NCL has the highest OSI and RRT on the aortic side. The asymmetric WSS distributions are mainly due to the presence of coronary arteries and the difference between flow through the left and right coronary arteries. For each leaflet, higher WSS is observed on the ventricular side and near the top edge, aligning with findings from previous FSI simulations of the Magna Ease valve (Sadipour, 2023).

It is worth noting that the area proximal to the valve product, primarily located in the sinus, has the highest OSI and RRT across the aortic root, indicating a potential trigger for thrombus formation in this region. While it is possible to directly estimate WSS and its related parameters on the aortic wall using high-resolution advanced imaging techniques, as demonstrated in a recently published study on the Magna Ease valve (Stephan et al., 2024), accurate quantification of WSS and mechanical stress on the valve leaflets still requires the integration of imaging and simulation tools. The boundary conforming FSI method adopted in this study offers a distinct advantage in this regard, allowing accurate determination of WSS and its related metrics that depend strongly on velocity gradient.

### 4.3 Limitations

The FSI model presented in this study has several limitations. First, the effect of turbulence during the systolic ejection phase was neglected, a flat velocity profile was specified at the LVOT inlet, the valve profile and the aortic wall were assumed to be rigid, and the mechanical behaviour of the valve leaflets was assumed to be isotropic. These assumptions could affect the predicted valve opening and closing dynamics, the surrounding haemodynamics and mechanical stress. Second, the coronary flow distributions were based on data from healthy adults (Kim et al., 2010), hence they represent a physiologically reasonable approximation for population-level simulations under baseline conditions rather than being patient-specific. For the model included in the current study, the diameters of the left and right coronary arteries (∼4.6 mm and 3.4 mm, respectively) approach the spatial resolution of the 4D flow MRI (3.1 × 3.1 × 3.1 mm^3^), making it impossible to directly extract the left and right coronary flowrates from the imaging data. Lastly, only one Magna Ease valve case was evaluated. Future work will aim to improve the workflow by incorporating a closed loop lumped parameter network to the inlet and outlets (Kim et al., 2009) or 3D patient-specific velocity profiles at the inlet (Pirola et al., 2017), adopting an anisotropic model for the valve leaflets, and considering the compliance of the valve profile and the aortic wall.

## 5 Conclusion

In this study, an FSI model of surgical BPAV incorporating non-Newtonian blood properties and coronary arteries was developed and applied to an Edwards Magna Ease valve in a patient-specific setting. The simulation results were compared with 4D flow MRI and relevant data in the literature, demonstrating good overall agreement with the patient’s *in vivo* data and other studies on the same product. The results further demonstrated that despite the symmetry of the valve leaflet geometry, WSS distributions on the leaflets were asymmetric, reflecting the influence of coronary flow. The FSI simulation workflow offers a promising tool for patient-specific assessment of aortic valve haemodynamics, and the results may help understand the link between haemodynamic metrics and valve leaflet thickening, calcification, or tearing of the prosthetic valve. Although the developed workflow was only applied to a 25 mm Magna Ease valve, the model can be easily adapted to simulate different BPAV products of different sizes.

## Data Availability

The raw data supporting the conclusions of this article will be made available by the authors, without undue reservation.
